# Isolation and characterization of antimutagenic components of *Glycyrrhiza aspera* against *N*-methyl-*N*-nitrosourea

**DOI:** 10.1186/s41021-016-0068-2

**Published:** 2017-01-06

**Authors:** Keiko Inami, Yusuke Mine, Jin Tatsuzaki, Chihiro Mori, Masataka Mochizuki

**Affiliations:** 1 Faculty of Pharmaceutical Sciences, Tokyo University of Science, Yamazaki 2641, Noda, Chiba 278-8510 Japan; 2 Tokiwa Phytochemical Co. Ltd, Sakura-shi, Japan

**Keywords:** Glyurallin A, Glyasperin B, Licoricidin, 1-Methoxyphaseollin, Licoisoflavone B

## Abstract

**Background:**

A powdered ethanolic extract of *Glycyrrhiza aspera* root exhibits antimutagenic activity against *N*-methyl-*N*-nitrosourea (MNU) based on the Ames assay with *Salmonella typhimurium* TA1535. The aim of this study was to identify the antimutagenic components of the powdered ethanolic extract of *G. aspera* root.

**Results:**

The powdered ethanolic extract of *G. aspera* root was sequentially suspended in *n*-hexane, carbon tetrachloride, dichloromethane, ethyl acetate, and ethanol, and each solvent soluble fraction and the residue were assayed for antimutagenic activity against MNU in *S. typhimurium* TA1535. The dichloromethane soluble fraction exhibited the highest antimutagenicity and was fractionated several times by silica gel chromatography. The fraction with the highest antimutagenic activity was further purified using HPLC, and the fractions were assayed for antimutagenicity against MNU in *S. typhimurium* TA1535. Finally, five components with antimutagenic activity against MNU were identified as glyurallin A, glyasperin B, licoricidin, 1-methoxyphaseollin, and licoisoflavone B.

**Conclusions:**

The five components were demonstrated to possess an antigenotoxic effect against carcinogenic MNU for the first time. It is important to prevent DNA damage by *N*-nitrosamines for cancer chemoprevention.

**Electronic supplementary material:**

The online version of this article (doi:10.1186/s41021-016-0068-2) contains supplementary material, which is available to authorized users.

## Background

Humans are exposed to endogenous and exogenous *N*-nitroso compounds [1]. Approximately 45–75% of the total human exposure to *N*-nitroso compounds is estimated to be due to in vivo synthesis [2]. Almost all tested *N*-nitroso compounds have carcinogenic activity in experimental animals [1]. Therefore, exposure to *N*-nitroso compounds is suspected to induce human cancer. Several epidemiological studies have demonstrated that the endogenous formation of *N*-nitroso compounds is correlated with the cancer incidence in humans [3–9]. Recently, the International Agency for Research on Cancer (IARC) has reported that the consumption of red meat and processed meat is carcinogenic, and this may be caused by *N*-nitroso compounds that form during meat processing or cooking [10].

*N*-Methyl-*N*-nitrosourea (MNU) is a DNA alkylating carcinogen that induces cancer in various organs, particularly the forestomach, brain and nervous system, in rodents [11]. MNU is produced by the nitrosation of creatinine or fermented foods at the gastric pH [12–15]. Additionally, MNU is formed by the nitrosation of methylurea with nitrite in guinea pig stomachs [16]. Therefore, for cancer chemoprevention, it is important to identify compounds that can inhibit mutagenicity induced by MNU.

Short-term bacterial mutation assays, such as the Ames assay, are an effective screening tool for the identification of various mutagenic or antimutagenic compounds in complex materials [17]. The assay has advantages as an inexpensive and flexible screening method that provides preliminary information related to antimutagenesis. There are many reports about the antimutagenicity of edible plants; however, the inhibitory effects against MNU mutagenesis are less well studied [18, 19].

*Glycyrrhiza* root has long been used worldwide as an herbal medicine and natural sweetener [20–22]. The genus *Glycyrrhiza* (Leguminosae) consists of about 30 species including *G. glabra*, *G. uralensis*, *G. inflata*, *G. aspera, G. korshinskyi and G. eurycarpa* [23]*.* In Japanese pharmacopeia, only *G. glabra* and *G. uralensis* are permitted to be used as licorice and licorice powder, and the other *Glycyrrhiza* species can be used as raw materials of licorice extract [23]. *Glycyrrhiza* has a reported chemopreventive effect based on its anticarcinogenesis and antimutagenesis toward both indirect-acting and direct-acting mutagens [24–29]; however, the inhibitory effects against MNU mutagenesis have not been studied in detail.

In our previous study, a powdered ethanolic extract of *G. aspera* root decreased MNU-induced mutagenicity in a preliminary antimutagenic screen using the Ames assay [30]. The aim of this study was to identify the antimutagenic components of the powdered ethanolic extract of *G. aspera* root.

## Methods

### General experimental procedures

The reaction progress was monitored using thin-layer chromatography (TLC) on silica gel 60 F_254_ (0.25 mm, Merck, Darmstadt, Germany). Column chromatography was performed using silica gel 60 (0.04–0.063 mm, Merck). Melting points were determined using a Yanaco (Tokyo, Japan) micro-melting-point apparatus without correction. HPLC was performed using an EYELA Preparative LC system [VSP-3050 pump, UV-9000 spectrometric detector, LiChrosorb RP-18 column (10 μm, 25 mm × 300 mm)] (Tokyo Rikakikai Co. Ltd., Tokyo, Japan) and a Shimadzu LC system [LC-6 AD pump, SPD-20A UV spectrometric detector, Mightysil RP-18 column (5 μm, 20 mm × 250 mm)] (Kyoto, Japan). The NMR spectra were recorded with a JEOL JNM-LA400 spectrometer (Tokyo, Japan). The chemical shifts were expressed in ppm, downfield from TMS. The mass spectra were collected using a JEOL JMS-SX102A mass spectrometer (Tokyo, Japan).

### Reagents

Sodium ammonium hydrogen phosphate tetrahydrate was purchased from Merck (Darmstadt, Germany). Bacto agar and Bacto nutrient broth were obtained from Becton Dickinson Microbiology Systems (Sparks, USA). MNU were obtained from Toshin Gousei (Tokyo, Japan). Other reagents were purchased from Wako Pure Chemical Industries (Osaka, Japan). A powdered ethanolic extract of *G. aspera* (China) root was kindly provided by Tokiwa Phytochemical Co. Ltd. (Chiba, Japan).

### Preparation of a powdered ethanolic extracts of *Glycyrrhiza aspera* root

A root of *G. aspera* (100 g) was refluxed with 95% ethanolic aqueous solution (1000 mL) for 1 h, and the mixture was filtered with suction. The residue was refluxed again with 95% ethanolic aqueous solution (1000 mL) for 1 h, and the mixture was filtered with suction. The combined filtrates were concentrated under reduced pressure and vacuum dried to a constant weight, and finally a brown powder was obtained.

### Fractionation of the powdered ethanolic extract of *Glycyrrhiza aspera* root based on solubility in organic solvents

The powered ethanolic extract of *G. aspera* root (10 g) was added to hexane (100 mL) and stirred for 10 min. The supernatant was filtered with suction. The stirring and filtration of the residue was repeated twice. Sequentially, the residue was suspended in carbon tetrachloride (100 mL × 3), dichloromethane (100 mL × 3), ethyl acetate (100 mL × 3), and ethanol (100 mL × 3) following the same procedure. The organic solvent portions were removed organic solvent by rotary evaporator and the residue was dried in vacuo. The whole extraction procedure was repeated twice; the organic portions and residue were combined. Finally, hexane soluble fraction (62 mg), carbon tetrachloride soluble fraction (880 mg), dichloromethane soluble fraction (15.6 g), ethyl acetate soluble fraction (11.4 g), ethanol soluble fraction (700 mg) and the residue (1.7 g) were obtained from the powdered ethanolic extract of *G. aspera* root (30 g). Recovery of the weight was 101%.

### Isolation of antimutagenic compounds from the dichloromethane soluble fraction

The dichloromethane soluble fraction was chromatographed on a silica gel, eluted with 5% methanol-CH_2_Cl_2_, 3% methanol-CH_2_Cl_2_, 1% methanol-CH_2_Cl_2_, 10% ethyl acetate-CH_2_Cl_2_, and later separated on an RP-18 column by preparative HPLC and eluted with 80% methanol in water (see the Additional file 1). Five peaks representing active components were purified using HPLC and characterized by comparing their spectroscopic (NMR and MS) properties with literature values.

### Bacterial mutation assay

The antimutagenic effect of each plant extract was assayed according to the Ames method using the plate-incorporation protocol [31, 32]. Dr. T. Nohmi (National Institute of Health Sciences, Tokyo, Japan) kindly provided the *S. typhimurium* TA1535.

A solution of MNU (1.5 μmol/50 μL DMSO) was added to a test tube and supplemented with 0.1 M sodium phosphate buffer (pH 7.4, 0.5 mL), a solution (50 μL) with various concentrations of fraction, and a culture of the *S. typhimurium* TA1535 (0.1 mL), and the solution was thoroughly mixed. Then, Top Agar (2 mL) was added, and the mixture was poured onto a minimal-glucose agar plate. The revertant colonies were counted after incubation at 37 °C for 44 h. Each sample was assayed using duplicate plates. The results are expressed as the mean number of revertant colonies per plate. Plates with neither MNU nor plant extract were considered negative controls. MNU (1.5 μmol/50 μL) resulted in 1470 ± 70 colonies. All of the tested plates were microscopically examined for thinning or the absence of a background lawn and/or presence of microcolonies, which are considered indicators of toxicity induced by the test material. Neither MNU nor plant extracts displayed toxicity to *S. typhimurium* TA1535 under the conditions of the antimutagenicity test.

Mutagenic activity in the presence of extracts is expressed as the percentage of mutagenicity (% = Rs/R × 100), where Rs is the number of his^+^ revertants/plate for plates exposed to MNU and plants extracts, and R is the number of his^+^ revertants/plate of MNU. The number of spontaneous revertants was subtracted beforehand to give Rs and R. Thus, the mutagenicity of MNU in the absence of plant extracts was defined as 100% MNU mutagenicity.

### Cytotoxicity test

Toxicity assays under the same conditions as those used for the Ames test were performed to determine the maximum concentrations of plant extracts that could be added without exerting toxic effects on the bacteria used in the Ames test. A solution of MNU (1.5 μmol/50 μL DMSO) was added to a test tube, and supplemented with 0.1 M sodium phosphate buffer (pH 7.4, 0.5 mL), each solution of plant extract (50 μL), and a culture of *S. typhimurium* TA1535 (0.1 mL). A portion of the mixture was diluted 10^5^ times in 1/15 M PB. The diluted solution (200 μL) was supplemented with histidine-free Top Agar (2 mL) and poured on a Nutrient Broth agar plate. The colonies were counted after incubation at 37 °C for 20 h. Each sample was assayed using duplicate plates. A substance was considered cytotoxic when the bacterial survival was less than 80% of that observed in the negative control. The mutation frequency was estimated as the number of mutants per 10^7^ surviving bacterial cells [31, 32].

## Results and discussion

### Identification of antimutagenic components from a powdered ethanolic extract of *Glycyrrhiza aspera* root

**Fig. 1:**
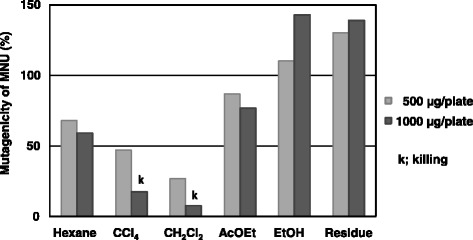
Effect of each soluble fraction on MNU-induced mutagenicity in *S. typhimurium* TA1535

A powdered ethanolic extract of *G. aspera* root was sequentially suspended in *n*-hexane, carbon tetrachloride, dichloromethane, ethyl acetate, and ethanol. Each soluble fraction and the residue were assayed for inhibitory effects against MNU mutagenesis in *Salmonella typhimurium* TA1535 (Fig. 1).

**Fig. 2:**
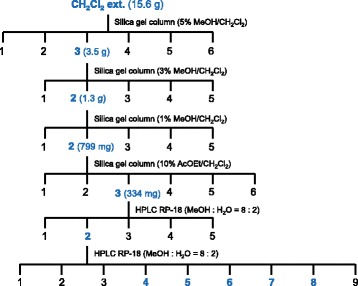
Diagram of the separation procedure for the dichloromethane soluble fraction

The dichloromethane soluble fraction showed the highest antimutagenic activity, and was fractionated using a silica gel column and preparative high-performance liquid chromatography (HPLC), and its antimutagenic components were identified. The fractionation and mutagenicity assay were conducted repeatedly (Fig. 2 and the Additional file 1). In each fractionation step, the recoveries of weights were more than 89%.

**Fig. 3:**
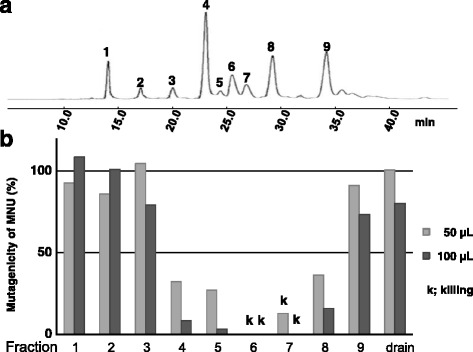
**a** HPLC diagram of Fr. 3-2-2-3-2; **b** Effect of each fraction from Fr.3-2-2-3-2 on MNU-induced mutagenicity in *S. typhimurium* TA1535

Finally, the fraction (Fr.3-2-2-3-2) with the highest antimutagenic activity was separated into fractions 1–9 using HPLC (Fig. 3a). Those fractions were tested for mutagenicity against MNU, and the compounds from peaks 4–8 each inhibited MNU-induced mutagenicity (Fig. 3b)

**Fig. 4:**
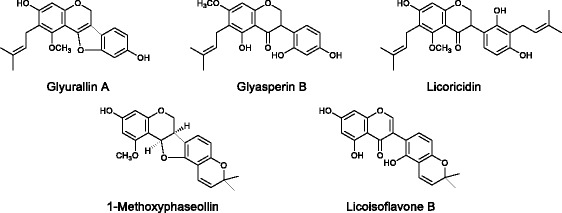
Structures of antimutagenic agents from the powdered ethanolic extract of *Glycyrrhiza aspera* root

The compounds from fractions 4–8 were each analysed using mass spectrometry and ^1^H nuclear magnetic resonance spectroscopy, and five antimutagenic compounds were identified, i.e., glyurallin A [33], glyasperin B [34], licoricidin [35, 36], 1-methoxyphaseollin [37], and licoisoflavone B [38], by comparing their spectroscopic properties with literature values (Fig. 4).

Glyurallin A was a pterocarpene and 1-methoxyphaseollin was a pterocarpan. Glyasperin B, licoricidin, and licoisoflavone B were an isoflavanone, an isoflavan, and an isoflavone, respectively. A phenolic hydroxyl group is a common in five isolated compounds with antimutagenicity.

Flavonoids are well-known antimutagens based on the results of Ames assays [39, 40], whereas pterocarpenes and pterocarpans are not well-known for their antimutagenicity.

### Inhibitory effect of licoricidin from the powdered ethanolic extract of *Glycyrrhiza aspera* root on MNU-induced mutagenicity

**Fig. 5:**
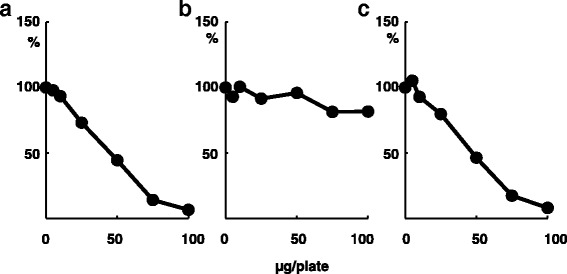
Mutagenicity (**a**), survival rate (**b**), and mutation frequency (**c**) of licoricidin on MNU-induced mutagenicity in *S. typhimurium* TA1535

Licoricidin (peak 6) did not show any revertant colonies by cytotoxicity (Fig. 3b). For the antimutagenicity assay, it was necessary to determine the concentration at which the viability of the tester strain did not decrease. Licoricidin reduced revertant colonies induced by MNU in *S. typhimurium* TA1535 (Fig. 5a) without cytotoxicity at the maximum concentration of

100 μg/plate (Fig. 5b). To assess the precise antimutagenic potency of licoricidin, mutation frequency was calculated by dividing the number of mutants with the surviving fraction of bacteria (Fig. 5c). These data clearly showed that licoricidin possessed antimutagenic activity against MNU in *S. typhimurium* TA1535.

The antimutagenic activity of the *G. glabra* extract against ethyl methanesulfonate (EMS) is reportedly attributed to glabrene [41]. In our study, glabrene was not isolated from the fraction with the highest mutagenic activity. The difference between the isolated compounds in antimutagenic activity for the powdered ethanolic extract of *G. aspera* root probably reflects differences in the mechanism of action between MNU and EMS. MNU reacts mainly via an S_N_1 mechanism and efficiently alkylated both nitrogens and oxygens in DNA. EMS, which reacts predominantly via an S_N_2 mechanism, alkylates the nitrogens at the DNA bases and produced little alkylation of the oxygens in DNA bases [42, 43]. Thus, the MNU is far more mutagenic than EMS. Additionally, the main bioactive components were different between *G. glabra* and *G. aspera* [23].

In vitro*, N*-nitrosamine formation is inhibited by phenolic compounds, ascorbic acid, thiols, and metals [18, 19]. Mutagenesis by direct-acting mutagens can be reduced or prevented in several ways; MNU can be decomposed to non-mutagenic products via antimutagens, or reactive mutagenic products from MNU can react with antimutagens before reaching DNA. It is also possible to induce repair enzymes in the *Salmonella* strain [44].

A number of known flavonoids (such as genistein etc) possess significant antimutagenic activity [40, 45, 46]; however, the detailed mechanism for the antimutagenicity has not been completely established. The inhibitory effect on the mutagenicity of direct-acting mutagens is probably caused by a chemical reaction between the mutagen and antimutagen. The inhibitory effect of phenolic acid results from the scavenging action on an electrophilic decomposition product of MNU [47]. Hung et al have reported that hydroxylated flavonoids showed antimutagenic activity toward benzo[*a*]pyrene 7,8-diol-9,10-epoxide by direct interaction with the 7,8-diol-9,10-epoxide [48]. Therefore, we assumed that the antimutagenic mechanisms of the isolated compounds were similar to that of phenolic acid or hydroxylated flavonoids. Furthermore, MNU treatments are reported to induce not only DNA alkylation but also increase intracellular ROS level [49–51], and then the antimutagenicity was partly attributed to its radical scavenging potency of flavonoids [52–54]. We are working to elucidate the antimutagenic mechanisms for the isolated compounds against MNU.

Antimutagens and anticarcinogens in the diet are suggested to be highly effective for cancer prevention [44, 55, 56]. The intake of medicinal and edible plants that include antimutagens may play a role in improving human health.

## Conclusions

It is important to prevent DNA damage by *N*-nitrosamines for cancer chemoprevention. In this study, five components with antimutagenic activity against MNU from a powdered ethanolic extract of *G. aspera* root were identified as glyurallin A, glyasperin B, licoricidin, 1-methoxyphaseollin, and licoisoflavone B. To the best of our knowledge, this report describes the first demonstration of the antigenotoxic effects of these components against carcinogenic MNU.

## Additional file

Additional file 1: Fractionations of antimutagenic compounds from dichloromethane soluble fraction. (DOCX 3675 kb)

## Abbreviation

MNU: *N*-Methyl-*N*-nitrosourea
