# Supplementary material for: Isolation and characterization of antimutagenic components of Glycyrrhiza aspera against N-methyl-N-nitrosourea
Source: Genes Environ. 2017 Jan 6;39:5. doi: 10.1186/s41021-016-0068-2 (PMC5217204; doi:10.1186/s41021-016-0068-2)
Supplement: Additional file 1: — Fractionations of antimutagenic compounds from dichloromethane soluble fraction. (DOCX 3675 kb) [file 41021_2016_68_MOESM1_ESM.docx]

Isolation and characterization of antimutagenic components of *Glycyrrhiza aspera* against *N*-methyl-*N*-nitrosourea

Keiko Inami^1,*^, Yusuke Mine^1^, Jin Tatsuzaki^2^, Chihiro Mori^2^, Masataka Mochizuki^1^

1 Faculty of Pharmaceutical Sciences, Tokyo University of Science, Noda-shi, Japan

2 Tokiwa Phytochemical Co. Ltd, Sakura-shi, Japan

Contents

Effect of Fr.1–6 from dichloromethane fraction on MNU-induced mutagenicity in *S. typhimurium* TA1535 (Figure S1) S1

Effect of Fr.3-1–3-5 from Fr.3 on MNU-induced mutagenicity in

*S. typhimurium* TA1535 (Figure S2) S2

Effect of Fr.3-2-1–3-2-5 from Fr.3-2 on MNU-induced mutagenicity in

*S. typhimurium* TA1535 (Figure S3) S3

Effect of Fr.3-2-2-1–3-2-2-6 from Fr. 3-2-2 on MNU-induced mutagenicity in

*S. typhimurium* TA1535 (Figure S4) S4

Fractionation of Fr. 3-2-2-3 by preparative HPLC (Figure S5) S5

Effect of Fr.3-2-2-3-1–3-2-2-3-5 from Fr.3-2-2-3 on MNU-induced mutagenicity in *S. typhimurium* TA1535 (Figure S6) S6

Antimutagenic activity of licoricidin (peak 6) against MNU in *S. typhimurium* TA1535 (Table S1) S7

**Isolation of antimutagenic compounds from dichloromethane soluble fraction**

The dichloromethane soluble fraction (15.5 g) was fractionated to No. 1–6 by silica gel column (400 g, 8.0 × 15.0 cm) eluted with 5% methanol-CH_2_Cl_2_; Fr.1 (2.7 mg), Fr.2 (1.7 g), Fr.3 (3.5 g), Fr. 4 (3.0 g), Fr.5 (3.7 g), Fr.6 (3.9 g). Recovery of the weight was 101 %.

Each of Fr.1–Fr.6 and the dichloromethane soluble fraction (before fractionation) were prepared at concentration of 500 and 1000 μg/50 μL methanol, and the solutions were tested for mutagenicity induced by MNU (1.5 μmol/50 μL DMSO) (Figure S1).

Fraction No.

1

2

3

4

5

6

CH_2_Cl_2_

Fraction No.

Fraction No.

Fraction No.

Fraction No.

Fraction No.

Figure S-1 Effect of Fr.1–6 fraction from the dichloromethane soluble fraction on MNU-induced mutagenicity in *S. typhimurium* TA1535

Fraction No.

Fr.3 (3.47 g) was fractionated to fractions 3-1–3-5 by silica gel column (30 g, 4.0 × 4.3 cm) eluted with 3% methanol-CH_2_Cl_2_; Fr. 3-1 (1.5 g), Fr. 3-2 (1.3 g), Fr. 3-3 (0.6 g), Fr. 3-4 (13 mg), Fr. 3-5 (20 mg). Recovery of the weight was 98 %.

Each of Fr.3-1–Fr.3-5 and Fraction 3 (before fractionation) were prepared at concentration of 250 and 500 μg/50 μL methanol, and the solutions were tested for mutagenicity induced by MNU (1.5 μmol/50 μL DMSO) (Figure S2).

Fraction No.

Figure S-2 Effect of Fr.3-1–3-5 from Fr.3 on MNU-induced mutagenicity in *S. typhimurium* TA1535

Fr.3-2 (1.26 g) was fractionated to fractions 3-2-1–3-2-5 by silica gel column (75 g, 4.0 × 10.5 cm) eluted with 1% methanol-CH_2_Cl_2_; Fr. 3-2-1 (5.8 mg), Fr. 3-2-2 (799 mg), Fr. 3-2-3 (210 mg), Fr. 3-2-4 (97 mg), Fr. 3-2-5 (138 mg). Recovery of the weight was 99 %.

Each of Fr.3-2-1–Fr.3-2-5 and Fraction 3-2 (before fractionation) were prepared at concentration of 50, 100 and 200 μg/50 μL methanol, and the solutions were tested for mutagenicity induced by MNU (1.5 μmol/50 μL DMSO) (Figure S3).

Fraction No.

Figure S-3 Effect of Fr.3-2-1–3-2-5 from Fr.3-2 on MNU-induced mutagenicity in *S. typhimurium* TA1535

Fr.3-2-2 (770 mg) was fractionated to fractions 3-2-2-1–3-2-2-6 by silica gel column (40 g, 3.0 × 10 cm) eluted with 10% ethyl acetate-CH_2_Cl_2_; Fr. 3-2-2-1 (7.2 mg), Fr. 3-2-2-2 (234 mg), Fr. 3-2-2-3 (334 mg), Fr. 3-2-2-4 (69 mg), Fr. 3-2-2-5 (18 mg), Fr. 3-2-2-6 (26 mg). Recovery of the weight was 89 %.

Each of Fr.3-2-2-1–Fr.3-2-2-6 and Fraction 3-2-2 (before fractionation) were prepared at concentration of 50, 100 and 200 μg/50 μL methanol, and the solutions were tested for mutagenicity induced by MNU (1.5 μmol/50 μL DMSO) (Figure S4).

Fraction No.

Figure S-4 Effect of Fr.3-2-2-1–3-2-2-6 from Fr. 3-2-2 on MNU-induced mutagenicity in *S.typhimurium* TA1535

Fr. 3-2-2-3 (10 mg) was separated on a LiChrosorb RP-18 column (25 mm × 300 mm, 10 μm, Phenomenex), flow rate 7.0 mL/min, with 80% methanol in water as eluent, detected by UV absorption at 254 nm; Fr. 3-2-2-3-1 (1.4 mg), Fr. 3-2-2-3-2 (6.4 mg), Fr. 3-2-2-3-3 (0.9 mg), Fr. 3-2-2-3-4 (1.8 mg), Fr. 3-2-2-3-5 (1.6 mg). Recovery of the weight was 114 % (Figure S5).


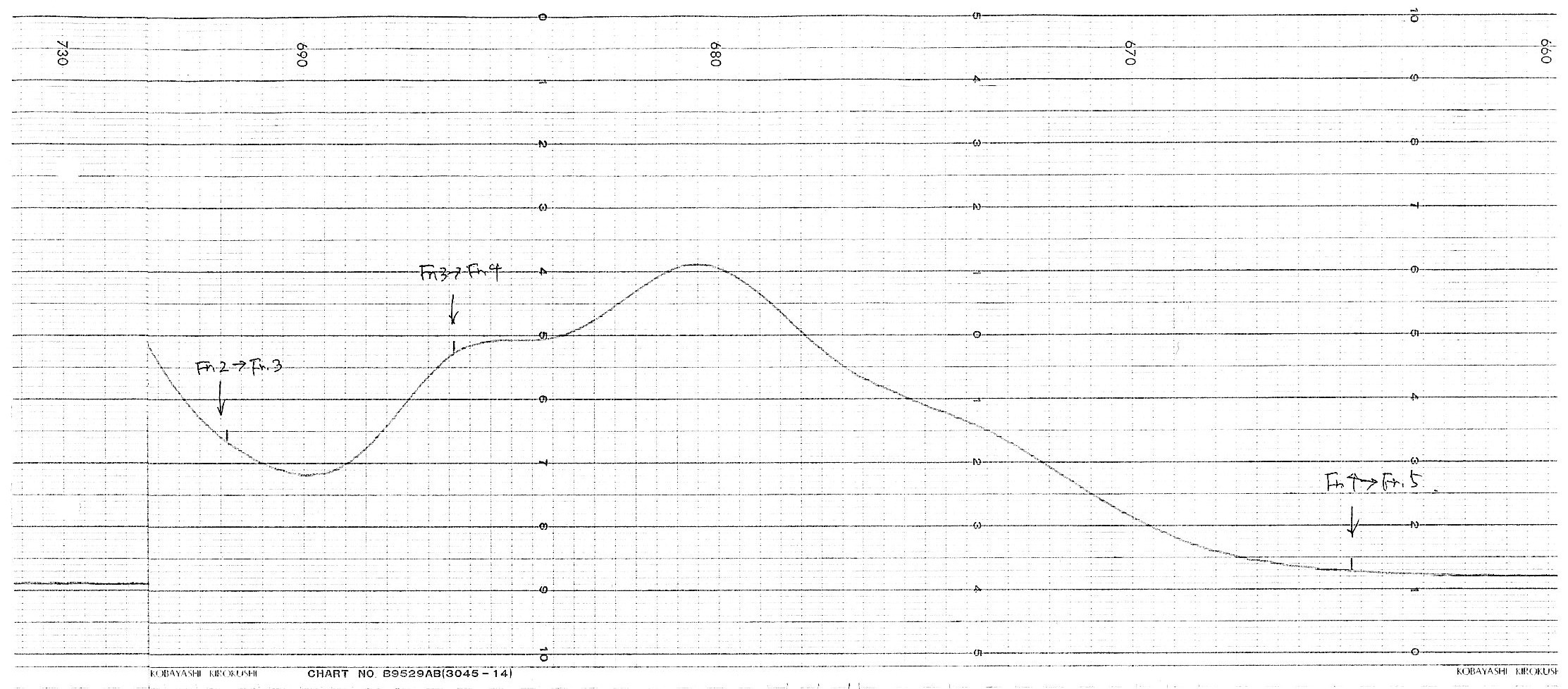

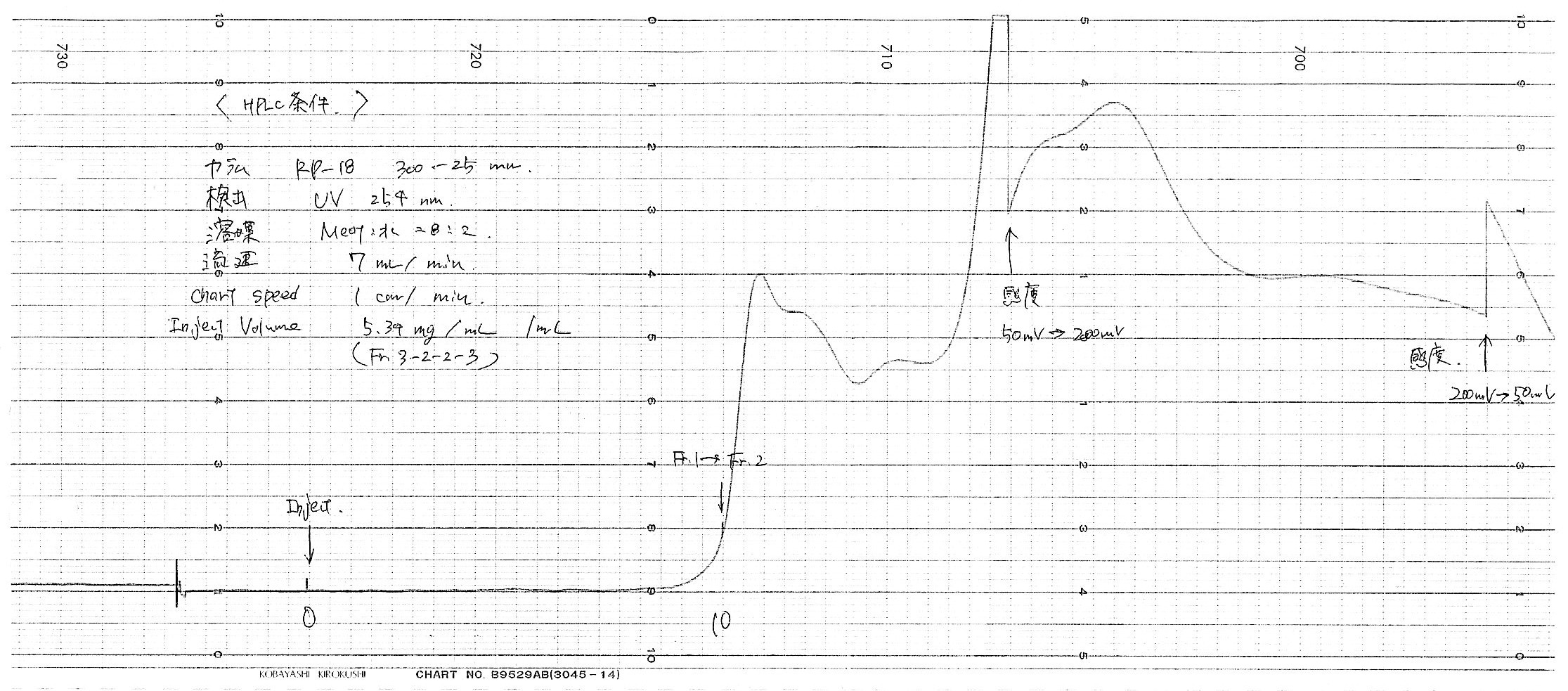

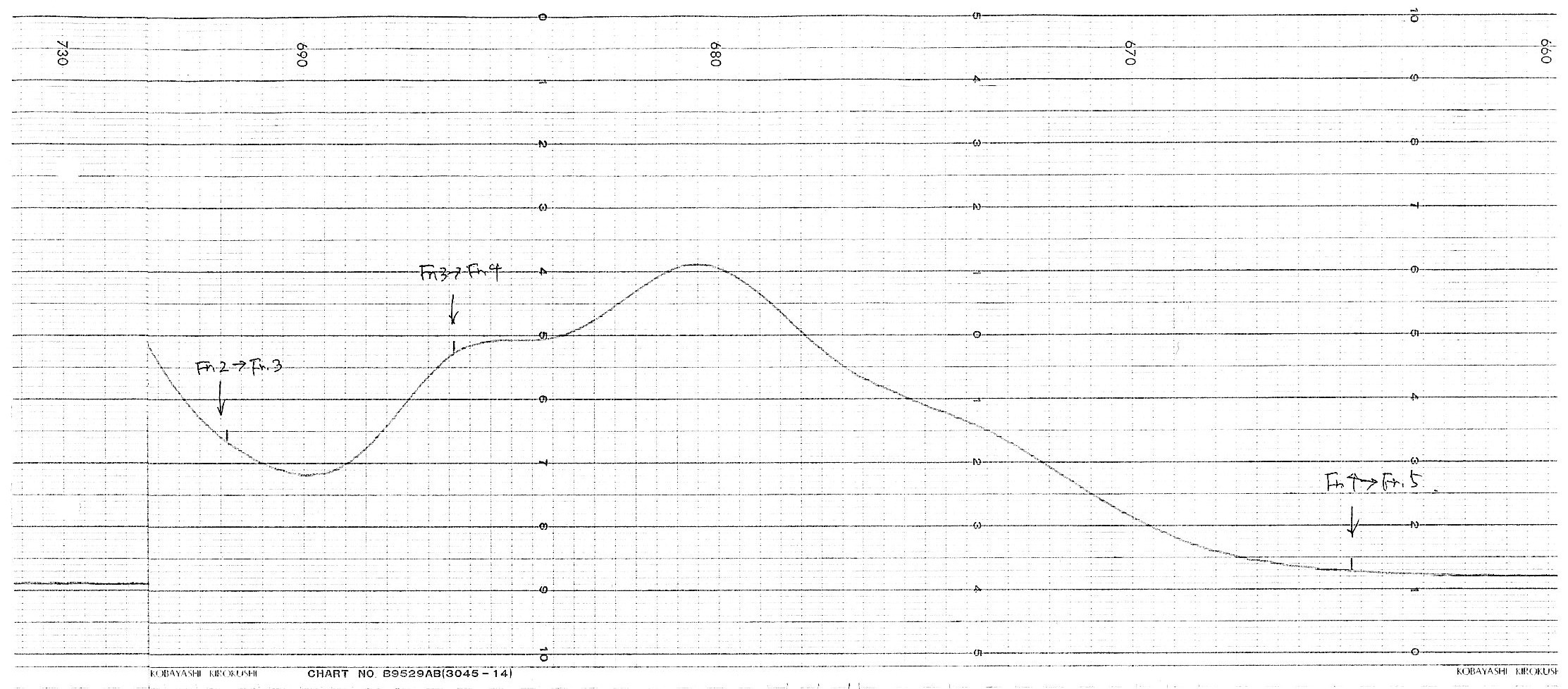


**Fr.1**

**Fr.2**

**Fr.3**

**Fr.4**

**Fr.5**

Figure S-5 Fractionation of Fr. 3-2-2-3 by preparative HPLC

Each of Fr.3-2-2-3-1–Fr.3-2-2-3-5 and Fraction 3-2-2-3 (before fractionation) were prepared at concentration of 50, 100 and 200 μg/50 μL methanol, and the solutions were tested for the mutagenicity induced by MNU (1.5 μmol/50 μL DMSO) (Figure S6).

Fraction No.

Figure S-6 Effect of Fr.3-2-2-3-1–3-2-2-3-5 from Fr. 3-2-2-3 on MNU-induced mutagenicity in *S. typhimurium* TA1535

Fr. 3-2-2-3-2 (76.1 mg) was separated on a Mightysil RP-18 column (20 mm × 250 mm, 5 μm, Kanto Chemical), flow rate 5.0 mL/min, with 80% methanol in water as eluent, detected by UV absorption at 254 nm; peak 1 (not determined), peak 2 (0.09 mg), peak 3 (not determined), peak 4 (not determined), peak 5 (0.06 mg), peak 6 (1.25 mg), peak 7 (0.20 mg), peak 8 (0.02 mg), peak 9 (0.03 mg), drain (1.24 mg) (Figure 3). Recovery of the weight was 107 % (Figure 3).

Some of the fractions could not be weighed, and then the whole eluent of each fraction was dissolved in 200 μL methanol, and the methanol solution (50 μL and 100 μL) were tested for the mutagenicity induced by MNU (1.5 μmol/50 μL DMSO) (Figure 3).

Table S1 Antimutagenic activity of licoricidin (peak 6) against MNU in *S. typhimurium* TA1535

| Concentration | Revertants/plate | Survival (%) | | MF |
| --- | --- | --- | --- | --- |
| (µg/plate) | ±SE | Colonies  ±SE | Survival rate (%) | (%) |
| 0 | 1104 ± 205 | 489 ± 51 | 100.0 | 100.0 |
| 5 | 1080 ± 133 | 455 ± 13 | 92.9 | 105.3 |
| 10 | 1032 ± 10 | 492 ± 0 | 100.6 | 92.9 |
| 25 | 807 ± 32 | 448 ± 18 | 91.6 | 79.8 |
| 50 | 491 ± 56 | 469 ± 11 | 95.9 | 46.4 |
| 75 | 156 ± 42 | 398 ± 27 | 81.4 | 17.4 |
| 100 | 73 ± 3 | 400 ± 21 | 81.7 | 8.1 |
| 150 | 39 ± 12 | 333 ± 14 | 68.1 | 5.2 |
| DMSO | 14 ± 0 | 453 ± 19 | 92.6 |  |
